# Impact of comprehensive genomic profiling on the diagnosis and clinical management of malignant mesenchymal tumours

**DOI:** 10.3389/pore.2025.1612065

**Published:** 2025-03-12

**Authors:** Anna Beáta Csepregi, Eszter Papp, Imola Adamik, Erzsébet Csernák, Helga Engi, Zsófia Küronya, Edina Soós, Zsombor Melegh, Erika Tóth

**Affiliations:** ^1^ Department of Surgical and Molecular Pathology, Centre of Tumour Pathology, National Institute of Oncology, Budapest, Hungary; ^2^ Department of Urogenital Tumours and Clinical Pharmacology, National Institute of Oncology, Budapest, Hungary; ^3^ National Tumour Biology Laboratory, National Institute of Oncology, Budapest, Hungary

**Keywords:** next generation sequencing, comprehensive genomic profiling, targeted therapy, sarcoma, mesenchymal tumours

## Abstract

Comprehensive genomic profiling (CGP) is becoming an increasingly important tool in the clinical management of different tumours, but there is still very limited data available on its usefulness from a therapeutic point of view in mesenchymal tumours. Between January 2022 and September 2024, we performed CGP analysis with means of Oncomine Comprehensive Assay Plus (OCAplus) on 94 malignant mesenchymal tumours. The analysis covered more than 500 unique genes for single-gene and multigene biomarker insights, including tumour mutational burden (TMB) and homologous recombination deficiency (HRD). Genomic DNA and total RNA were extracted from formalin-fixed paraffin-embedded tissue blocks. Twenty-four out of 94 patients (25.5%) had potentially actionable alterations: 17 (18%) had specific genetic alterations suitable for targeted therapies, 4 (4.2%) had a high TMB (>10 mut/Mb), and 5 (5.3%) had a high HRD score >15). One additional patient had *BRCA1* mutation, but the HRD score was low. Three patients received targeted therapy: one patient with a *CDK4*-amplified tumour (dedifferentiated liposarcoma) received CDK4 inhibitor therapy, two patients with angiosarcoma showing high TMB received immune checkpoint inhibitor therapy, and one patient with a uterine leiomyosarcoma and high HRD score received PARP inhibitor therapy. In addition, two patients with malignant phyllodes tumours received multi-thyrosine kinase inhibitor therapy. In three cases, there was refinement or reassignment of the diagnosis, based on the CGP findings. Our results demonstrate that CGP can provide useful additional information and can be beneficial in the clinical management of patients with mesenchymal tumours.

## Introduction

Malignant mesenchymal tumours are a heterogeneous tumour group, currently there are more than 70 histological subtypes designated in the WHO classification [1], and it can be often difficult to make an accurate diagnosis. Therefore, it is recommended that these cases are assessed in centralised centres with appropriate immunohistochemistry and molecular laboratories. Most localised sarcomas are treated by surgical resection with adjuvant or neoadjuvant radiotherapy in some cases. The role of adjuvant chemotherapeutic treatment, apart from a few exceptions, is not well established. In advanced, metastatic disease most patients have a poor prognosis with the current systemic therapies, hence the increasing need to develop new therapeutic options [2, 3]. In these cases, comprehensive genomic profiling (CGP) by next-generation sequencing (NGS) can potentially help to identify pharmacologically actionable mutations [4]. Here, we summarise the results of the CGP studies performed on mesenchymal tumours at the National Institute of Oncology, Budapest between January 2022 and September 2024.

## Materials and methods

**FIGURE 1:**
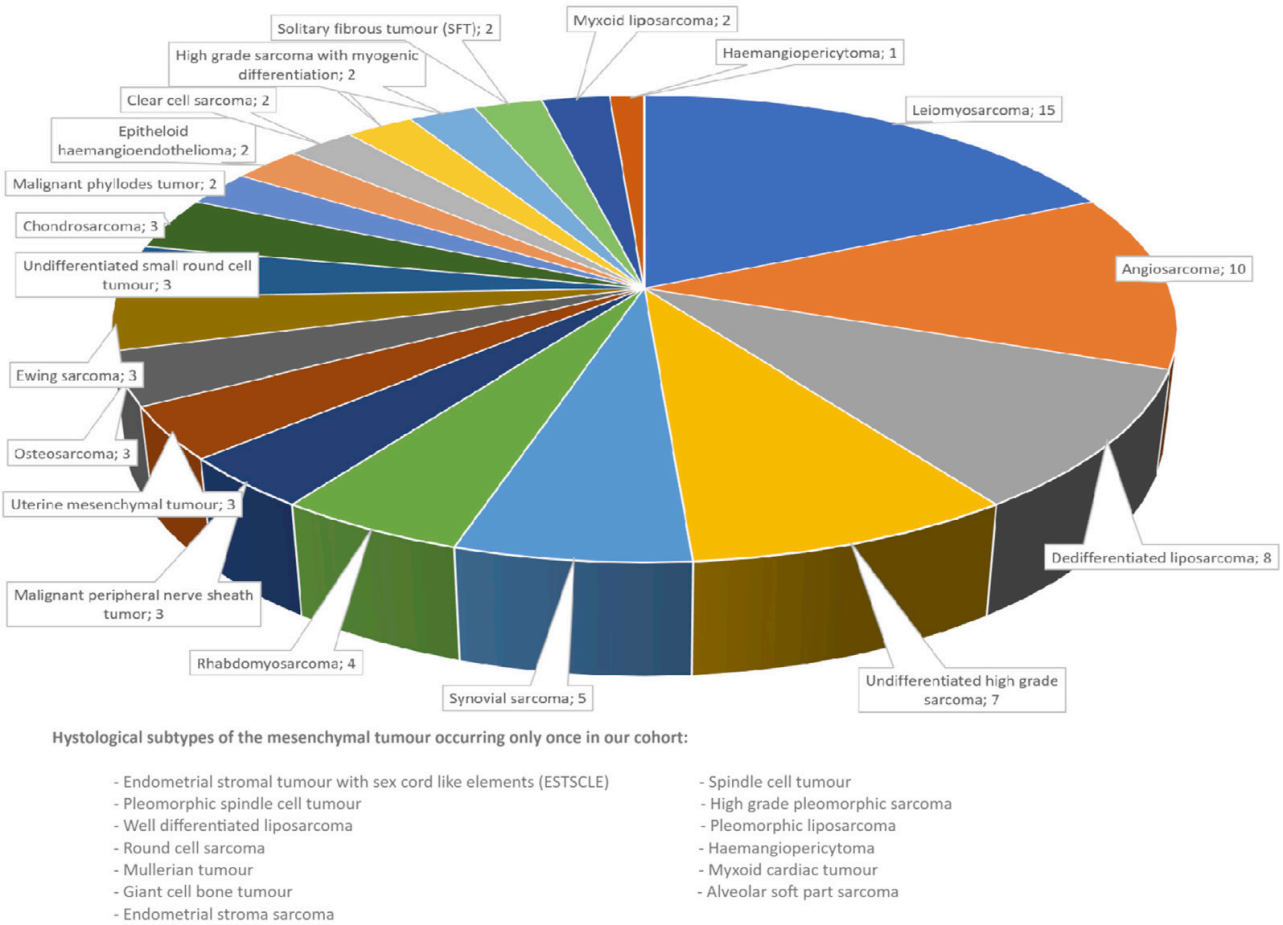
Histological types of the mesenchymal tumours assessed.

Between January 2022 and September 2024, we performed 415 CGP analyses overall, where the number of tests requested has been increasing each year. Out of the 415 CGP analyses 94 were performed on mesenchymal tumours, representing 32 histologies (Figure 1). The cohort was not restricted to soft tissue sarcoma, but also included cases that represented pure mesenchymal tumours of parenchymal organs (Müllerian sarcoma and phyllodes tumour). DNA and total RNA were extracted from formalin-fixed paraffin-embedded tissue blocks. Libraries were prepared using the Ion Chef™ System with Ion 540™ Chips (Thermo Fisher Scientific, Waltham, MA, United States) according to the manufacturer’s instructions with a DNA input of approximately 4 ng and an RNA input of 5.7 ng. Sequencing was performed using an Ion S5™ Plus Sequencer (Thermo Fisher Scientific, Waltham, MA, United States). We used Ion Reporter™ Software (v. 5.18) (Thermo Fisher Scientific, Waltham, MA) for data analysis. We used Oncomine Comprehensive Assay Plus (OCA Plus) RNA GX as the analysis workflow for the samples. The analysis covered more than 500 unique genes for single-gene and multigene biomarker insights, including microsatellite instability status (MSI), homologous recombination deficiency (HRD) and tumour mutational burden (TMB). High HRD was defined as a score >15, high TMB as >10 mut/Mb. Only those mutations were recorded which were categorized as pathogenic or likely pathogenic at the workflow.

A proportion of the tumours had also been subjected to preliminary small-panel studies (monogenic COBAS KRAS and BRAF/NRAS mutation tests, Oncomine Comprehensive Assay (160 genes), Oncomine Focus Assay and Precision Assay (50 genes) or FusionPlex Pan Solid Tumor v2 NGS panels). Indication for CGP followed the European Society for Medical Oncology (ESMO)guideline: the procedure was initiated at the Molecular Genetics and Rare Cancer Tumour Board when the therapeutic options had been exhausted but the patient was still in good condition. All patients were at clinical status ECOG 0 or 1 [5].

## Results

**FIGURE 2:**
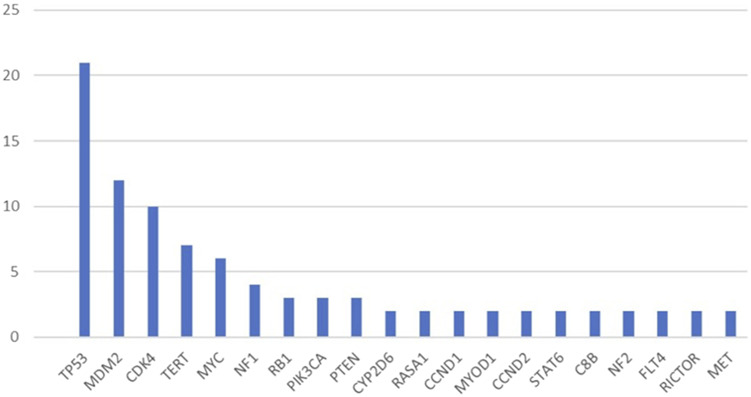
Most commonly identified pathogenic mutations (mutations detected more than once).

Ninety-four mesenchymal tumours were investigated, all of which were advanced and/or metastatic diseases. Out of the 94 patients, 55 were female and 39 were male, the median age was 52 years. The detected mutations are summarised in Figure 2 and Table 1.

**TABLE 1:** Pathogenic mutations and actionable fusions, which occurred only once in our cohort.

Other pathogenic mutations identified									
CIC	USP9X	FANCI	DPYD	SMAD4	KLC1	LATS	ARID1A	HLA-B	KLF5
MECOM	APC	ATRX	SPEN	ZFHX3	KDR	GNAS	POLE	SETBP1	MUTYH
YAP1	EIF1AX	FGF23	MCL1	PIK3R2	FGF4	FGF19	FGF3	EMSY	MEN1
ERBB4	NQO1	ETV6	FANCG	IDH2	KDM5C	BMPR2	CTFC	SLX4	JAK3
ATM	H3-3A	IKBKB	CTNNB1	NAB2	RNASEH2B	IDH1	NRAS	TRIO	FAM135B
NTRK3	PDGFRB	WT1	AKT1	IL7R	ESR1	ROS1	SMARCB1	EP300	FHFR3
MAP3K4	MPL	TRPV5	ARID5B						
Identified actionable fusions (number of cases)									
NTRK3 [1] - High grade sarcoma with myogenic differentiation					RET [1] - Chondrosarcoma				

There were only 16 cases (17%), where no pathogenic mutation was identified by OCAplus, while in 57% of the cases there was more than one pathogenic mutation. The most common tumour type with no additional pathogenic mutation was synovial sarcoma (Table 2).

**TABLE 2:** Histological subtype and TMB value of cases where no pathogenic mutations were identified by OCAplus.

Histological subtype	TMB
Synovial sarcoma [3]^a^	0 1.9 3.79
Leiomyosarcoma [2]	0 0.95
Angiosarcoma	0
GIST	0.94
Round cell sarcoma	0.95
Rhabdomyosarcoma NOS	0.96
Ewing sarcoma^a^	1.9
Alveolar rhabdomyosarcoma^a^	1.9
Myxoid cardiac tumour	3.8
High grade sarcoma with myogenic differentiation	5.7
Undifferentiated small round cell tumour	5.7
Uterine mesenchymal tumour	5.74

^a^ Diagnostic pathogenic gene fusions were detected by FISH and/or FusionPlex Pan Solid Tumour v2 NGS method.

Brackets = number of cases, if occurred more than once.

Twenty-four out of the 94 patients had potentially actionable specific genetic or biomarker alterations. Out of these 25 patients, 17 had specific genetic alterations suitable for targeted therapy: eight patients had *MDM2* and *CDK4* amplified dedifferentiated liposarcomas (one of these patients had high HRD score as well) 1 patient had *MDM2* and *CDK4* amplified well differentiated liposarcoma and 1 patient had sclerosing rhabdomyosarcoma with *MDM2* amplification, 1 patient had parosteal osteosarcoma with *MDM2* and *CDK4* amplification. A pathogenic IDH2 mutation was identified in 1 patient with osteoblastic osteosarcoma. There was one undifferentiated small round cell tumour with a pathogenic *BRAF V600E* mutation. One additional patient with uterine leiomyosarcoma had a pathogenic *BRCA2* mutation, but the HRD score was low. One patient with high grade myxoid liposarcoma had a pathogenic *PIK3CA* mutation identified. Two additional patients with malignant phyllodes tumours had pathogenic *PDGFRB* and *PIK3CA* mutations, respectively).

Four patients had a high TMB (>10 mut/Mb), and 5 had a high HRD score (>15) (Table 3). Those patients who had a low TMB, the average TMB was 3.51 mut/Mb. The average TMB for those with no pathogenic mutation detected was 2.01 mut/Mb, while it was 3.74 mut/Mb for those with at least one pathogenic mutation detected (Table 2). Those patients who had a low HRD score, the average HRD score was 4.37. MSI was identified in two cases: one had a high TMB (high grade sarcoma with myogenic differentiation), while the other had a high HRD score (undifferentiated high grade sarcoma).

**TABLE 3:** Actionable mutations and biomarkers detected in different histological subtypes (In brackets are the number of cases of each histological subtype).

Potentially targetable pathogenic mutations	Tumours with high TMB	Tumours with high HRD score
*Dedifferentiated liposarcoma* [6]*:* MDM2 and CDK4 amplification	*Angiosarcoma* [2]	*Mullerian sarcoma* [1]
*Parosteal osteosarcoma* [1]*:* MDM2 and CDK4 amplification *Osteoblastic osteosarcoma* [1]*:* IDH2 mutation	*Pleomorphic spindle cell tumour* [1]	*Undifferentiated high grade sarcoma* [1]
*Malignant phyllodes tumour* [2]*:* PIK3CA and PDGFRB mutation	*High grade sarcoma with myogenic differentiation* [1]	*High grade sarcoma with myogenic differentiation* [1]
*Undifferentiated small round cell tumour* [1]*:* BRAF V600E mutation		*Uterine leiomyosarcoma* [1]
*Sclerosing rhabdomyosarcoma* [1]*:* MDM2 amplification		*Dedifferentiated liposarcoma* [1]
*Uterine leiomyosarcoma* [1]*:* BRCA2 mutation		
*High grade myxoid liposarcoma* [1]*:* PIK3CA		
*Well differentiated liposarcoma* [1]*:* MDM2 and CDK4 amplification		

Based on these results, six patients received targeted therapy: one patient with a CDK4-amplified tumour (dedifferentiated liposarcoma) received CDK4 inhibitor, two patients with angiosarcoma and high TMB received immune checkpoint inhibitor therapy. All these 3 patients are currently alive (follow up time: 22 months, 12 and 11 months, respectively). One patient, with uterine leiomyosarcoma and high HRD score received PARP inhibitor therapy, died of the disease. Both patients with malignant phyllodes tumours and pathogenic *PDGFRB* and *PIK3CA* mutations received multi-thyrosine kinase inhibitor therapy but died of the disease.

For 3 patients, there was refinement or reassignment of the diagnosis: *MYOD1* mutation (sclerosing rhabdomyosarcoma), *MDM2* and *CDK4* amplification (change of diagnosis from biphenotypic sinonasal carcinoma to parosteal osteosarcoma), and *VHL* mutation (change of the diagnosis from spindle cell tumour with mesenchymal character to sarcomatoid renal cell carcinoma).

Kinase fusion also represents a potentially actionable target is sarcomas. We detected kinase fusion in 2 cases (3.2%) of the tumours: *RET* fusion in 1 case, and *NTRK3* fusion in another case.

A summary of the small panel NGS studies on mesenchymal tumours carried out in our institution in the same period (between January 2022 and September 2024) is shown in Tables 4, 5. During this period, 232 small, targeted panel NGS studies were performed on mesenchymal tumours, including 105 gastrointestinal stromal tumours (GIST), comprising the largest proportion of this tumour group. As the small panel assessment was considered sufficient in most GISTs, there were only 2 cases, where subsequent CGP was performed. Of the 94 patients assessed with CGP, 68 had a preliminary small panel NGS assessment.

**TABLE 4:** Preliminary monogenic and small panel NGS examinations on mesenchymal tumours (total: 94).

Preliminary examination	Number of cases
Oncomine Comprehensive Assay (160 genes)	16
Oncomine Focus Assay an Precision Assay (50 genes)	6
Monogenic panels (KRAS, NRAS, BRAF)	3
FusionPlex Pan Solid Tumour v2 RNA-based fusion test	1
No preliminary examination	68

**TABLE 5:** Monogenic and small panel studies carried out on mesenchymal tumours in our institute between January 2022 and September 2024.

Small panel	2022	2023	2024 (from January to September)
Oncomine Comprehensive Assay (160 genes)	30	56	18
Oncomine Focus Assay and Precision Assay (50 genes)	36	32	27
Monogenic tests	3	5	1
RNA-based fusion test	-	5	10

## Discussion

Our results, based on the genomic profiling of 94 patients, demonstrate that CGP can provide useful additional information and can be helpful in the clinical management of patients with mesenchymal tumours.

Genomic profiling can be beneficial in the refinement of the diagnosis as well as finding potentially targetable genomic alterations. Sarcoma diagnostics is considered a notoriously complex part of histopathology, requiring specialized knowledge, hence most difficult cases are often referred to specialist centres. In our cohort, 3.2% of the cases had their diagnosis altered or refined based on the CGP results. This is much lower, than reported in the literature (around 10%) - this is probably due to the fact that most of the cases included were reported by specialist soft tissue pathologists [7, 8]. In one case, there was refinement of diagnosis, aiding the specialist classification of a rhabdomyosarcoma into sclerosing subtype. A more important implication is a change of diagnosis from sarcoma to carcinoma or *vice versa*. In our cohort, where there was a change of diagnosis from biphenotypic sinonasal carcinoma to parosteal osteosarcoma in a head and neck case, the correct diagnosis could potentially be reached at the time of initial diagnosis by performing MDM2 FISH or immunohistochemistry. In the case of sarcomatoid renal cell carcinoma, the correct diagnosis could be only reached based on the detected *VHL* mutation, as no epithelial component was present on histology. There was one case of undifferentiated small round cell tumour with a pathogenic *BRAF V600E* mutation, this raises the possibility that this case could represent a dedifferentiated malignant melanoma, but this cannot be substantiated due to lack of other specific markers and no clinical data of previous melanocytic lesion.

The most common pathogenic mutation in our cohort was in *TP53* at a rate of 22.3%, which is in line reported in the literature [8]. Pathogenic *TP53* mutation is reported to be associated with poor prognosis and is most commonly seen in pleomorphic sarcomas [6]. The second most common alteration affected MDM2 and *CDK4* genes, this is due to the relative prevalence of dedifferentiated liposarcomas in our cohort. *MDM2* and *CDK4* amplification is a potential therapeutic target in these tumours with ongoing Phase I and II trials of CDK4 and/or MDM2 inhibitors, but there are no established treatment options developed yet based on these genetic alterations [9]. Of note, TP53 alteration could lead to resistance to CDK4/6 and MDM2 inhibitors, respectively [10].

Performing CGP also allows us to assess specific biomarkers, including HRD, TMB and MSI [11, 12]. PARP inhibitors have been demonstrated as an effective treatment in specific tumour groups with alterations in DNA damage repair pathways or with high HRD signature [8]. In our cohort, there was high HRD in 5 out of the 94 cases (5.3% of tumours), with all the 5 cases detected representing different histologies (Table 3). Immune checkpoint inhibitor monotherapy so far has had disappointing results in sarcoma treatment and there is lack of reliable biomarkers that could be implemented in clinical practice. In contrast to high HRD tumours, where all tumours represented different histologies, there was over-representation of angiosarcomas in the high TMB cohort. Overall, there were 4 high TMB tumours (4.2%), out of which 2 were angiosarcomas, which comprised 20% of all angiosarcomas examined (2 out of 10). High prevalence of TMB has been reported in angiosarcomas, especially in cutaneous angiosarcoma, and the high mutational burden can be secondary to etiological ultraviolet (UV) light exposure [13]. MSI-High (MSI-H) status was identified in only two cases (2.1%). MSI-H is reported at a very low proportion in malignant mesenchymal tumours, it is below 1% [8, 14]. In our study 2.1% of the tumours were MSI-H, which is still very low, therefore we do not think that routine mismatch repair (MMR) or MSI testing is advisable in sarcoma diagnostics. However, the combined prevalence of high HRD, high TMB and MSI-H is relatively high, indicating them as a potential biomarker of PARP inhibitor or immune checkpoint inhibitor therapy in sarcoma treatment.

We found actionable alterations in 23.8% of the patients, which is at the lower end of the range previously reported in the literature in different sarcoma subtypes [8, 15]. This can be due to the fact that in our practice small NGS panels are performed more often than CGP, and CGP is performed only in those cases where the small panel is not informative enough, or the disease progression indicates assessment of a wider range of potentially targetable alterations. During the period covered by our analysis, 232 small panel NGS analyses were performed on mesenchymal tumours at our institution and only 68 had a subsequent CGP analysis. Consequently, in our cohort of 94 patients, there were only 26 patients who had no previous NGS analysis. This especially applies to GIST, where actionable genomic alteration is very common; in our cohort there were only 2 GIST included, previously shown to be wild type GIST with smaller panel.

There were 16 cases (17%) where no pathogenic mutations were detected by OCAplus (Table 2). This rate is somewhat higher than that reported in the literature by FoundationOne and Tempus NGS methods (10.3%) [8, 16]. The most common histological subtypes with no detectable pathogenic mutation in our study were synovial sarcoma (3 out 5 cases) followed by leiomyosarcoma (2 out of 14 cases). In addition to synovial sarcomas, there were two other fusion-associated sarcomas among these 16 cases: a Ewing sarcoma and an alveolar rhabdomyosarcoma. In these cases, although no pathogenic mutation was deceted by OCAplus, diagnostic gene fusions were previously identified by FISH and/or FusionPlex Pan Solid Tumour v2 NGS method. Concerning the TMB, the average TMB of these cases was lower than seen in the rest of our cohort (2.01 vs 3.74 mut/Mb), suggesting a lower level of genomic instability in these tumours. This is in keeping with previous findings, which also found a lower TMB in fusion associated sarcomas [8]. The over-representation of fusion-associated sarcomas in the mutation-negative cohort may also partially explain our study’s higher rate of negative results. The focus of OCA Plus panel is on targetable alterations, hence it does not detect most sarcoma specific gene fusions. In contrast, CGP panels specifically designed for sarcoma testing, such as FoundationOne Heme would detect these diagnostic fusions.

There is also some limited data available on whole genome sequencing (WGS) of sarcomas, which is a more comprehensive method for tumour genome analysis [17, 18]. Whole genome analysis has led to the detection of a higher number of pathogenic mutations, and a lower proportion of cases with no detected pathogenic mutations compared to our study (4.5%) [17]. While the focus of OCA Plus panel is on pathogenic mutations with known therapeutic implications, whole exome sequencing (WES) may detect additional pathogenic mutations, but with limited clinical relevance [17]. Although the role of these additional pathogenic mutations in sarcoma tumorigenesis is currently uncertain, this additional data could offer valuable insights into tumour biology in the future [17].

In 4.8 percent of cases there was a change in medical treatment because of our CGP results, which represents only a small proportion of potential actionable targets and is less than reported in other studies [8, 16]. Most targeted therapeutical regimes require significant resources from a healthcare provider and accessibility of a treatment option can greatly vary between countries depending on their economical resources. Performing CGP analysis on mesenchymal tumours also incurs extra costs in the diagnostic process, however its cost has significantly reduced recently, and continue to be decreasing. Our results demonstrate that CGP can provide useful additional information and can be beneficial in the clinical management of a significant proportion of patients with mesenchymal tumors. Consequently, as the costs are decreasing, CGP should become the first-line tool in identifying therapeutic opportunities for the benefit of the patient. In summary, we can say that comprehensive genomic studies are increasingly important for accurate histological classification of soft tissue tumours. In addition, in a tumour group with relatively narrow therapeutic options, CGP reveals possible effective targeted therapies in many cases and will significantly aid the search for further effective therapies and inclusion of patients in clinical trials.

## Data Availability

The original contributions presented in the study are included in the article/supplementary material, further inquiries can be directed to the corresponding authors.
